# In Silico Integrated Analysis of Genomic, Transcriptomic, and Proteomic Data Reveals QTL-Specific Genes for Bacterial Canker Resistance in Tomato (*Solanum lycopersicum* L.)

**DOI:** 10.3390/cimb45020090

**Published:** 2023-02-06

**Authors:** Ibrahim Celik

**Affiliations:** 1 Department of Agricultural and Livestock Production, Cal Vocational School of Higher Education, Pamukkale University, Denizli 20700, Turkey; icelik@pau.edu.tr; Tel.: +90-546-573-58-18; 2 Plant Genetics and Agricultural Biotechnology Application and Research Center (PAU BIYOM), Pamukkale University, Denizli 20160, Turkey

**Keywords:** *Clavibacter michiganensis* subsp. *michiganensis*, disease resistance, QTL mapping, candidate gene identification, wild tomato species

## Abstract

Bacterial canker of tomato, caused by *Clavibacter michiganensis* subsp. *michiganensis* (*Cmm*), is a devasting disease that leads to significant yield losses. Although QTLs originating from three wild species (*Solanum arcanum*, *S. habrochaites*, and *S. pimpinellifolium*) were identified, none of the QTLs was annotated for candidate gene identification. In the present study, a QTL-based physical map was constructed to reveal the meta-QTLs for *Cmm* resistance. As a result, seven major QTLs were mapped. Functional annotation of QTLs revealed 48 candidate genes. Additionally, experimentally validated *Cmm* resistance-related genes based on transcriptomic and proteomic studies were mapped in the genome and 25 genes were found to be located in the QTL regions. The present study is the first report to construct a physical map for *Cmm* resistance QTLs and identify QTL-specific candidate genes. The candidate genes identified in the present study are valuable targets for fine mapping and developing markers for marker-assisted selection in tomatoes for *Cmm* resistance breeding.

## 1. Introduction 

Tomatoes (Solanum lycopersicum L.) are one of the most produced vegetables in the world due to their high adaptation capacity and economic value. While tomato plays a significant role in agriculture, biotic and abiotic stresses have a negative impact on tomato production [1,2]. Bacterial canker caused by *Clavibacter michiganensis* subsp. *michiganensis* (*Cmm*) is one of the devastating diseases of tomatoes [3,4]. Although copper-based chemical treatments are used to control the disease, natural resistance genes are considered the most effective method [4,5]. 

Wild tomato species such as *Solanum arcanum*, *Solanum habrochaites*, and *Solanum pimpinellifolium* were found to be tolerant to *Cmm* [6,7,8]. Mapping studies demonstrated that *Cmm* resistance was a quantitatively inherited trait. Thus, several QTLs originating from wild tomato species were mapped in the tomato genome. An early QTL mapping study performed by Sandbrink et al. [9] identified five QTLs on chromosomes 1, 6, 7, 8, and 10 using backcross populations derived from *S. arcanum* (LA 2157). Later, the same wild species were used to map three QTLs on chromosomes 5, 7, and 9 [6]. Moreover, two QTLs originating from *Solanum habrochaites* (LA407) (Rcm 2.0 and Rcm 5.1 located on chromosomes 2 and 5, respectively) were identified [7,10,11]. Finally, 18 QTLs originating from *S. pimpinellifolium* were mapped on chromosomes 1, 2, 7, 8, and 12, among which the QTL on chromosome 7 was reported as the major locus [8].

Transcriptomic and proteomic approaches were used to provide insight into the molecular mechanisms of *Cmm* resistance [12,13,14]. A microarray representing 9254 genes was used to identify DEGs (Differentially Expressed Genes) in pathogen-inoculated *S. lycopersicum* genotypes (Rio Grande; Nr mutant and its background lines Pearson and Ailsa Craig). As a result, 122 genes were differentially expressed, 11 up-regulated, and four down-regulated genes were verified using qRT-PCR. Furthermore, the study reported that the ethylene-insensitive mutant *Never ripe* (*Nr*) had reduced disease symptoms [13]. Balaji and Smart [15] reported that overexpression of Snakin-2 (SN2) and extensin-like protein (ELP) caused enhanced tolerance for *Cmm*. Afterwards, a cDNA-AFLP method was performed in *Cmm* inoculated *S. arcanum* and *S. habrochaites* wild tomato species. As a result, a total of 403 genes were found to be differentially expressed [14]. Afterwards, the SUMO E2 gene, identified by Lara-Avila et al. [14] was confirmed to provide resistance against *Cmm* when down-regulated by tomato mottle virus-induced gene silencing [16]. In addition, a proteomic approach was implemented in lines that had Rcm 2.0 and Rcm 5.1 *Cmm* resistance QTLs. As a result, 47 proteins were analyzed using ESI-MS/MS (electrospray ionization-mass spectrometry), and 26 differentially expressed proteins were annotated [12]. Despite these genomic, transcriptomic, and proteomics-based studies, the molecular function of the QTLs for the *Cmm* resistance mechanism is not known. Thus, the present study aimed to annotate the QTLs to identify candidate genes conferring resistance for *Cmm* by performing physical mapping of the QTLs and experimentally validated genes in the tomato genome. 

## 2. Material and Methods 

### 2.1. Physical Mapping of QTLs for Cmm Resistance

The present study aimed to map QTLs in the tomato reference genome. Meta-QTL analysis was not performed due to the comparatively low resolution of the genetic maps. Thus, physical mapping of molecular markers flanking QTLs was performed. For the mapping, two approaches were used. Firstly, probe sequences of RFLP (Restriction Fragment Length Polymorphism) markers and the recent tomato genome assembly (SL.4.0) were downloaded from the SOL genomics network database (https://solgenomics.net/) (accessed on 10 January 2023). Probe sequences of RFLP markers from Sandbrink et al. [9] and Van Heusden et al. [6] were blasted to the tomato genome using command-line BLAST tools (BLAST+) such as blastn (Megablast) with default parameters [17]. Secondly, in silico PCR, using primer sequences from the respective publications [7], was performed using e-PCR software with parameters of Max mismatches 2, Max indels 2, and PCR product 100–400 [18]. Details of QTL studies are given in Table S1. A physical map was drawn using MapChart software [19].

### 2.2. Functional Annotation of QTLs for Disease Resistance Genes

ITAG4.1 gene model list located on mapped QTLs was extracted from the genome browser of the tomato genome assembly SL4.0 available in the SOL genomics network database (https://solgenomics.net/jbrowse_solgenomics/?data=data%2Fjson%2FSL4.0&loc=SL4.0ch02%3A49089537..50188947&tracks=maker&highlight=) (accessed on 10 January 2023). Functions of genes located in each QTL were determined based on ITAG4.0 annotation (https://solgenomics.net/ftp/tomato_genome/annotation/ITAG4.0_release/) (accessed on 10 January 2023).

### 2.3. Physical Mapping of Experimentally Validated Cmm Resistance Genes

Transcript sequences had a role in *Cmm* resistance based on transcriptomic data reported by Balaji et al. [13] were downloaded from the NCBI database. Spliced alignment of transcript sequences was performed using Splign software with default parameters provided in the NCBI genome workbench browser [20,21]. Protein sequences from the publications of Coaker et al. [12] and Lara-Ávila et al. [14] were downloaded from NCBI and UniProt protein databases for performing a tblastn search using default parameters. Details of transcriptomic and proteomic studies are given in Table S2. 

## 3. Results 

### 3.1. Physical Mapping of QTLs for Cmm Resistance

**Figure 1:**
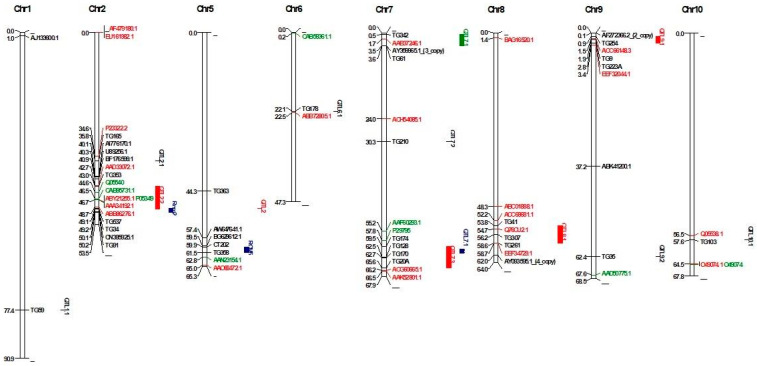
Physical map of QTLs and differentially expressed genes. Black-colored genes were reported by Balaji et al. [13], red-colored genes were reported by Lara-Ávila [14], and green-colored genes were reported by Coaker et al. [12].

QTLs originating from two wild tomato species (*S. arcanum* LA 2157) and *S*. *habrochaites* LA 407) were mapped on the genome based on markers that had a significant association with *Cmm* resistance. Firstly, QTLs originating from *S. arcanum* (LA 2157) were mapped based on a BLAST search of 18 RFLP probes located on chromosomes T1, T2, T6, T7, T8, T9, and T10 as reported by Sandbrink et al. [9]. The alignment identity of the probes ranged from 97.65% to 100%. As a result, a total of 18 RFLP probes were mapped on chromosomes these chromosomes (Table S3). Markers clustered in an interval of less than 10 Mb were considered single QTLs. As a result, 10 QTLs were mapped in the tomato genome. While four QTLs (QTL2.2, QTL7.3, QTL8.1, and QTL9.1) were mapped based on multiple probes, six QTLs (QTL1.1, QTL2.1, QTL7.1, QTL7.2, QTL9.2, and QTL10.1) were mapped based on a single probe (Table S4). For mapping of the QTLs originating from *S. arcanum* (LA 2157) reported by Van Heusden et al. [6], four RFLP probes found to be associated with *Cmm* resistance were mapped in the genome. Physical mapping of five probes on chromosomes T5, T7, and T9 revealed three QTLs. The QTL on chromosome T7 was mapped in an interval between 0.48 Mb (TG342) and 3.57 Mb (TG61), 3.08 Mb in size (Figure 1). The QTL on chromosome T9 was mapped in an interval between 0.85 Mb and 2.82 Mb, 1.96 Mb in size. TG363, linked to the QTL on chromosome T5, was mapped at position 44.33 Mb. QTLs on chromosome T7 and T9 were consensus QTLs identified by both studies [6,9].

QTLs originating from *S. habrochaites* LA 407 were mapped on chromosomes T2 and T5 using flanking makers [7,11]. As a result, the physical positions of two QTLs were identified. Rcm 2.0 was mapped in an interval between 49.09 Mb and 50.19 Mb, 1.10 Mb in size. Rcm 5.1 was mapped in an interval between 59.86 Mb and 61.46 Mb, 1.6 Mb in size (Figure 1).

Recently, *S. pimpinellifolium* was also reported as a resistance source for *Cmm* [8]. Thus, QTLs originating from this wild species were mapped on chromosomes T1, T2, T7, T8, and T12). The QTL on chromosome T7 had a significant effect and co-localized with the QTL originating from *S. arcanum* [6,8].

### 3.2. Functional Annotation of QTLs for Disease Resistance Genes

Physical mapping of markers linked to *Cmm* resistance revealed seven major QTLs for disease resistance on chromosomes T2, T5, T7, T8, and T9 (Table 1). The rest of the QTLs were excluded from analysis due to single marker matches, which might be unreliable. Two QTLs (mQTL2.2 and Rcm2.1) on chromosome 2 are located close to each other with a distance of 107.8 kb. mQTL2.2 was mapped in an interval between 43.0 Mb and 49 Mb (6.2 Mb in size). The mQTL2.2 interval contained 836 gene models, and the function of 540 gene models were determined and two genes (Solyc02g082740.1.1, Solyc02g084610.1.1, Solyc02g084600.4, and Solyc02g084610.1) were found with disease resistance function (Table S5). Rcm2.1 mapped at position 49.09 Mb to 50.19 Mb (1.1 Mb in size) had 139 gene models, and the functions of 124 gene models were determined. None of the gene models had disease-resistance functions. 

On chromosome T5, one major QTL (Rcm 5.0) originating from *S*. *habrochaites* was mapped at position 59.86–61.46 Mb (1.6 Mb in size). Rcm 5.0 had 140 gene models, and the function of 67 gene models was known. Although none of the gene models were related to disease resistance, four genes (Solyc05g051200.1.1, Solyc05g051310.1.1, Solyc05g050830.3, and Solyc05g050790.3) had ethylene-responsive factor functions that had a role in *Cmm* resistance [13] (Table S5). 

On chromosome 7, two QTLs (mQTL7.1 and mQTL7.3) were mapped in the genome [6]. mQTL7.1 was also reported by Sandbrink et al. [9], with 303 gene models and 11 gene models (Solyc07g006700.1.1, Solyc07g007740.1.1, Solyc07g007750.3, Solyc07g007730.4, Solyc07g007735.1, Solyc07g007710.4, Solyc07g007755.1, Solyc07g008373.1, Solyc07g008377.1, Solyc07g008375.1, and Solyc07g006710.2) had disease resistance functions among 213 gene models that had known function. Originating from *S. arcanum*, mQTL7.3, similar to mQTL7.1, was mapped at positions 59.48–65.63 Mb (Sandbrink et al. [9]. Additionally, mQTL7.3 also had QTL mapped at positions 60.29–61.49 Mb (1.2 Mb in size) originating from *S. pimpinellifolium* [8]. The QTL mQTL7.3 had 674 gene models. Although the function of 560 of these gene models is known, 14 gene models (Solyc07g056600.1.1, Solyc07g150139.1, Solyc07g053010.3, Solyc07g053020.3, Solyc07g049700.1, Solyc07g052785.1, Solyc07g052800.3, Solyc07g055620.2, Solyc07g055610.3, Solyc07g052770.2, Solyc07g055380.1, Solyc07g052790.3, Solyc07g052780.3, and Solyc07g055390.1) had a disease resistance function (Table S5). 

A meta-QTL, mQTL8.1, originating from *S. arcanum,* was mapped at positions 53.832322–58.596385 (4.76 Mb in size) on chromosome T8. The QTL contains 435 gene models, and the function of 397 gene models is known with one gene model (Solyc08g068360.1.1) annotated as an ethylene receptor and six models had disease resistance function (Solyc08g067380.1, Solyc08g074250.3, Solyc08g075980.2, Solyc08g075630.3, Solyc08g075640.4, Solyc08g076000.4).

Originating from *S. arcanum,* mQTL9.1 was mapped on chromosome T9 based on the QTL analysis reported by Sandbrink et al. [9] and van Heusden et al. [6]. The QTL mQTL9.1 contained 313 gene models, and the function of 282 gene models is known. While six genes (Solyc09g007010.1.1, Solyc09g007020.1.1, Solyc09g005950.3, Solyc09g006005.1, Solyc09g007010, and Solyc09g007020.1) had disease resistance function, two genes (Solyc09g009240.1.1 and Solyc09g005490.1.1) were seen as having ethylene signaling function (Table S5). 

### 3.3. Physical Mapping of Experimentally Validated Cmm Resistance Genes 

DEGs based on transcriptomic and proteomic studies were mapped to better understand the molecular mechanism of identified QTLs for *Cmm* resistance. Firstly, 15 genes (11 upregulated and four downregulated genes) during *Cmm* infection verified by qRT-PCR were mapped in the genome [13]. As a result, the genes mapped to chromosomes T1, T2, T4, T5, T7, T8, T9, T10, T11, and T12. Three genes coding Cys protease (AI776170.1), ERF/AP2 transcription factor (U89256.1), and pro-rich protein (BF176599.1) mapped at positions 40,102,846 bp, 40,311,718 bp, and 40,867,346 bp, respectively, were located close to mQTL2.2 based on the closest marker mapped at position 42.997178 Mb reported by Sandbrink et al. [9] (Table 2). Furthermore, peroxidase (AW647641.1) and chitinase (BG629612.1) genes were mapped close to Rcm 5.0 on chromosome T5. Distances between genes and Rcm 5.0 were 2.49 and 0.37 Mb for AW647641.1 and BG629612.1, respectively. For chromosome T7, an EIX receptor 1 gene (AY359965.1) was mapped at position 3,546,391 close to mQTL 7.1, reported by van Heusden et al. (1999). For chromosome T9, a Verticillium wilt resistance-related protein coding gene (AF272366.2) was mapped close to mQTL 9.1 reported by Sandbrink et al. [9] and van Heusden et al. [6] with a distance of 0.79 Mb (Figure 1). Details of QTL-specific genes are given in Table 2. 

In addition to microarray-based differentially expressed genes, 30 out of 33 DEGs identified by Lara-Ávila et al. [14] were mapped on the tomato genome. The percent identity of mapped proteins ranged from 54.67% to 100% (Table S6). As a result, a gene of Oxygen-evolving enhancer protein (P23322.2) was mapped at position 34,603,225 bp close to mQTL 2.1 (mapped position at 35.820075 Mb based on closest marker) reported by Sandbrink et al. [9], while a secretory peroxidase gene (AAD33072.1) was mapped close to mQTL2.2 with a distance 0.27 Mb, two RuBisCO (ABY21255.1 and AAA34192.1) and one mitochondrial processing peptidase-like gene (ABB86276.1) were mapped in a confidence interval of QTL2.2. For chromosome T6, a Per1-like family protein gene (ABB72805.) was mapped close to mQTL6.1 with a 0.37 Mb distance. For chromosome T7, a calmodulin-binding protein gene (AAB37246.1) was mapped in a confidence interval of mQTL7.1. For chromosome T8, two genes (ACC68681.1 and EEF34729.1 code vacuolar processing enzyme 2 and trehalose-6-phosphate synthase proteins, respectively) were mapped close to mQTL8.1, as reported by Sandbrink et al. [9], with distances 1.63 and 0.11 Mb for ACC68681.1 and EEF34729.1, respectively. For chromosome T9, a gene of cell division cycle protein (ACC66148.3) was mapped in a confidence interval of mQTL9.1, and other gene codes signal recognition particle protein (EEF32044.1) was mapped close to the QTL with a distance of 0.62 Mb. For chromosome T10, an endochitinase gene (Q05538.1) was mapped close to mQTL10.1 with 1.08 Mb (Figure 1).

DEGs identified in tomato lines had two *Cmm* resistance QTLs (Rmc2.1 and Rcm5.0) based on proteomic analysis that was mapped on the tomato genome. As a result, three genes (Q05540, CAB95731.1, and ABY21255.1 encoding endochitinase, allene oxide cyclase, and Ribulose-1,5-bisphosphate carboxylase/oxygenase small subunit, respectively) were mapped in confidence interval mQTL2.2 on chromosome T2. For chromosome T5, a dihydrolipoamide dehydrogenase precursor gene (AAN23154.1) was mapped close to Rcm5.0 with a distance of 1.36 Mb. Another gene (P29795) was mapped close to mQTL7.3 with a distance of 1.68 Mb (Figure 1). 

## 4. Discussion 

### 4.1. Physical Mapping of QTLs for Cmm Resistance

The first step of the development of pathogen-resistant tomato cultivars is the identification of resistance resources and mapping of the resistance genes or QTLs in the genome for the development of molecular markers. This approach was used for several bacterial, viral, and fungal diseases resistance controlled by single genes (reviewed by Foolad, and Panthee [22]. In contrast to these diseases, bacterial canker resistance is a quantitative trait, and the development of molecular tools for the disease is difficult. Although several QTL mapping studies were performed for *Cmm* resistance, none of the QTLs were used in tomato resistance breeding. Thus, combining all QTL data is essential for revealing the importance of identified QTLs and the determination of stable QTLs for a better understanding of the nature of resistance. A meta-QTL analysis is a commonly used approach in many species, including tomatoes [23,24,25]. The present study performed meta-QTL analysis for *Cmm* resistance for the identification of stable QTLs originating from three wild tomato species (*S. arcanum*, *S*. *habrochaites*, and *S. pimpinellifolium*) for the first time by physically mapping molecular markers linked to QTLs on the tomato genome [6,7,8,9]. As result, although single marker regression analysis (Kruskal-Wallis rank-sum test) was used by Sandbrink et al. [9] due to a limited number of markers, physical mapping of the markers revealed positions of 10 QTLs, which are valuable targets for marker development for *Cmm* resistance breeding in tomatoes. The physical positions of three QTLs originating from the same wild tomato species (*S. arcanum*) were determined on chromosomes T5, T7, and T9 [6]. Thus, both studies pointed out that QTLs on T7 and T9 were stable QTLs and valuable targets for developing molecular tools. Interestingly, the present study showed that a major QTL originating from *S. pimpinellifolium* co-localized with mQTL7.3. This finding may imply that *S. arcanum* LA 2157 and *S. pimpinellifolium* (GI.1554) may share common mechanisms for *Cmm* resistance. 

Physical mapping of QTLs (Rcm 2.1 and Rcm 5.0) originating from *S*. *habrochaites* was performed in the present study for the first time. The present study narrowed the confidence intervals of the QTLs to 2.05% and 2.45% of their respective chromosomes for Rcm 2.1 and Rcm 5.0, respectively, which is helpful for fine mapping of the candidate genes located in the QTLs. 

### 4.2. Functional Annotation of QTLs for Disease Resistance Genes 

One reason for the limited utilization of QTL data in marker-assisted selection is a lack of identified candidate genes in the QTLs that confer the QTL effect [26]. The present study aimed to determine the candidate genes in QTLs controlling *Cmm* resistance. The QTLs contained a reasonable number of gene models and the functions of most of the gene models were terminated. The percentage of genes that had known functions ranged from 47.86% to 91.26%. As result, a total of 48 genes had disease resistance or ethylene-related functions. Six of the identified genes (Solyc05g051200, Solyc05g051310, Solyc08g068360, Solyc09g009240, Solyc09g005490, and Solyc02g084610) were members of the ERF (Ethylene-Responsive Factor) gene family that regulates ethylene-inducible PR (pathogenesis-related genes) which have a function in pathogen attack [27,28]. The present study demonstrated the importance of ethylene in controlling resistance to *Cmm* in tomatoes. This is similar to the results of a study that reported that the ethylene insensitive mutant *Never ripe* (*Nr*) reduced disease symptoms of *Cmm* [13]. The rest of the genes (42 genes) were members of the pathogenesis-related protein family which have functions in pathogen resistance [29].

### 4.3. Physical Mapping of Experimentally Validated Cmm Resistance Genes 

Transcriptomics and proteomics are commonly used for the identification of DEGs during biotic and abiotic stresses to detect genes that mediate stress response. Two transcriptomic and one proteomic studies were performed on *S. lycopersicum* and *S. habrochaites* using microarray, cDNA-AFLP, and ESI-MS/MS methods. Several of the genes identified in these studies had *Cmm* resistance functions. The present study performed mapping of these genes on the tomato genome. As a result, 25 experimentally validated genes were mapped to *Cmm* resistance QTLs. QTL2.2 originating from *S. arcanum* had the largest number of genes (10 genes) followed by Rcm 5.0, QTL8.1, and QTL9.1 each had three genes. The results demonstrated that QTL.2.2 was the richest in terms of functional genes with the potential to provide resistance to *Cmm*. The present findings showed that QTL2.2 has a major role in *Cmm* resistance and is a good target with the other three QTLs (Rcm 5.0, QTL8.1, and QTL9.1) for the development of molecular tools for cultivar development. Most of the genes mapped in QTLs had disease-resistance functions as expected. Two QTLs (QTL2.1 and QTL2.2) had photosynthesis-related genes such as RuBisCO and Oxygen-evolving enhancer protein. This is expected because reduced photosynthesis enables plant cells to activate antimicrobial metabolites to control disease [14]. These QTLs had genes that directly function as a defense against fungus and bacteria, such as endochitinases that induce phytoalexin production [14]. Furthermore, Rcm 5.0 had genes related to carbon metabolism. This is expected because carbon metabolism was reduced during stress for cell survival [12]. The gene (U89256.1) coding ERF/AP2 transcription factors located in QTL2.2 also highlighted the role of ethylene in resistance for *Cmm* similar to results reported by [13].

In contrast to genes mapped in QTLs, some of the genes are mapped to other chromosomes. Possible explanations for this might be those minor effect loci were masked by more significant QTLs and the fact that the maps used in QTL studies had low resolution. Thus, significant improvement in QTL mapping for *Cmm* resistance is required for high-resolution genotyping and sensitive genomic approaches such as association mapping.

## 5. Conclusions

The present study performed mapping of QTLs originating from three wild tomato species (*S. arcanum*, *S*. *habrochaites*, and *S. pimpinellifolium*) to control *Cmm* resistance. As a result, a QTL-based physical map was constructed to reveal a more complete picture of the genomics of *Cmm* resistance. A total of seven major QTLs were identified originating from three wild tomato species on chromosomes T2, T5, T7, T8, and T9. In the present study, mQTL7.3 was identified as a consensus QTL for *S. arcanum* and *S. pimpinellifolium.* QTLs contained 48 genes with stress resistance functions. Furthermore, mapping of differentially expressed genes during *Cmm* infection revealed nine genes located within QTLs. Mapping of DEGs revealed the functional importance of QTL2.2 due to its having the highest number of genes (10 genes) differentially expressed during *Cmm* infection. Functional annotation of QTLs and mapping of DEGs reinforced the importance of the function of ethylene for *Cmm* resistance. These QTL-specific genes could be valuable targets for fine mapping and developing markers for direct marker-assisted selection in tomato *Cmm* resistance breeding. The present study showed that combining data from different omics technologies provided higher performance for candidate gene identification in plant genomics.

## Figures and Tables

**Table 1:** Physical map of meta-QTLs.

Meta-QTLs	Chromosome	Resistance Origin	Start (bp)	End (bp)	Size (Mb)	Flanking Markers	Reference
mQTL 2.2	2	* Solanum arcanum *	42,997,178	49,197,691	6.2	TG353-TG34	Sandbrink et al. [9]
Rcm 2	2	* S. habrochaites *	49,089,879	50,189,289	1.1.	TG537-TG91	Coaker and Francis [7]
Rcm 5.1	5	* S. habrochaites *	59,858,052	61,457,155	1.6	CT202-TG358	Coaker and Francis [7]
mQTL7.1	7	* Solanum arcanum *	484,118	3,565,619	3.08	TG342-TG61	Van Heusden et al. [6] and Sandbrink et al. [9].
mQTL7.3	7	* Solanum arcanum * and *S. pimpinellifolium*	59,483,951	65,632,413	6.15	TG174-TG20A	Sandbrink et al. [9]
mQTL8.1	8	* Solanum arcanum *	53,832,322	58,596,385	4.76	TG41-TG261	Sandbrink et al. [9]
mQTL9.1	9	* Solanum arcanum *	85,353	2816,764	2.73	TG254-TG223A	Van Heusden et al. [6] and Sandbrink et al. [9].

**Table 2:** Physical map positions of *Cmm* resistance genes.

Gene ID	Solyc Gene ID	Function	Map Position (bp)	Chromosome	QTL Position (Mb)	Distance to Closest QTL (Mb)	QTL	Reference
P23322.2	Solyc02g065400.3.1	Oxygen-evolving enhancer protein	34,603,225	T2	35.820075	1.22	QTL2.1	Lara-Ávila et al. [14]
AI776170.1	SGN-U580776	Cys protease	40,102,846	T2	42.997178	2.89	QTL2.2	Balaji et al. [13]
U89256.1	LOC544042 (*PTI5*)	Pti5, ERF/AP2 transcription factor	40,311,718	T2	42.997178	2.69	QTL2.2	Balaji et al. [13]
BF176599.1	No match	Pro-rich protein	40,867,346	T2	42.997178	2.13	QTL2.2	Balaji et al. [13]
Q05540	Solyc02g082930.3	PR-3 (CHIB_SOLLC Acidic 27 kDa endochitinase)	44,550,387	T2	42.997178	0	QTL2.2	Coaker et al. [12]
CAB95731.1	Solyc02g085730.3	Allene oxide cyclase	46,545,033	T2	42.997178	0	QTL2.2	Coaker et al. [12]
P05349	Solyc02g085950.3	Ribulose bisphosphate carboxylase small chain	46,721,911	T2	42.997178	0	QTL2.2	Coaker et al. [12]
AAD33072.1	Solyc02g080530.2	Secretory peroxidase	42,723,281	T2	42.997178	0.27	QTL2.2	Lara-Ávila et al. (2012)
ABY21255.1	No match	Ribulose-1,5-bisphosphate carboxylase/oxygenase small subunit	46,721,678	T2	42.997178	0	QTL2.2	Lara-Ávila et al. [14]
AAA34192.1	Solyc03g034220.3	Ribulose-1,5-bisphosphate carboxylase, a small subunit	46,730,615	T2	42.997178	0	QTL2.2	Lara-Ávila et al. [14]
ABB86276.1	No match	Mitochondrial processing peptidase-like	48,739,848	T2	42.997178	0	QTL2.2	Lara-Ávila et al. [14]
AW647641.1	EST307119	Peroxidase	57,368,022	T5	59.858052	2.49	rcm5	Balaji et al. [13]
BG629612.1	No match	Chitinase	59,492,036	T5	59.858052	0.37	rcm5	Balaji et al. [13]
AAN23154.1	Solyc05g053300.3	Dihydrolipoamide dehydrogenase precursor	62,819,021	T5	61.457155	1.36	rmc5	Coaker et al. [12]
ABB72805.1	No match	Per1-like family protein	22,480,227	T6	22.112109	0.37	QTL6.1	Lara-Ávila et al. [14]
AAB37246.1	No match	Calmodulin-binding protein	1,671,041	T7		0	QTL7.1	Lara-Ávila et al. [14]
AY359965.1	Solyc07g008620.1	EIX receptor 1	3,546,391	T7	3.565619	0.02	QTL7.1	Balaji et al. [13]
P29795	No match	Oxygen-evolving enhancer protein	5,780,491	T7	59.483951	1.68	QTL7.3	Coaker et al. [12]
ACC68681.1	No match	Vacuolar processing enzyme 2	52,199,457	T8	53.832322	1.63	QTL8.1	Lara-Ávila et al. [14]
Q76CU2.1	No match	PDR1 Pleiotropic drug resistance protein	54,718,024	T8		0	QTL8.1	Lara-Ávila et al. [14]
EEF34729.1	No match	Trehalose-6-phosphate synthase, putative	58,701,556	T8	58.596385	0.11	QTL8.1	Lara-Ávila et al. [14]
ACC66148.3	No match	Cell division cycle protein	154,958	T9		0	QTL9.1	Lara-Ávila et al. [14]
EEF32044.1	No match	Signal recognition particle protein, putative	3,438,392	T9	2.816764	0.62	QTL9.1	Lara-Ávila et al. [14]
AF272366.2	Ve1	Verticillium wilt disease R protein	58,717	T9	2.816764	0.79	QTL 9.1	Balaji et al. [13]
Q05538.1	Solyc10g055810.2	Basic 30 kDa endochitinase	56,492,366	T10	57.572715	1.08	QTL10.1	Lara-Ávila et al. [14]

## Data Availability

Not applicable.

## Supplementary Materials

The following supporting information can be downloaded at: https://www.mdpi.com/article/10.3390/cimb45020090/s1, Table S1: Details of studies performed QTL mapping for *Cmm* resistance; Table S2: Studies identified differentially expressed genes during *Cmm* infection; Table S3: Physical mapping of RFLP probe sequences associated with *Cmm* resistance reported by Sandbrink et al. [9]; Table S4: Table S4. QTLs identified based on physical map of RFLP probes reported by Sandbrink et al. [9]; Table S5: Functional Annotation of QTLs for Disease Resistance Genes. Table S6: Tblastn search of protein sequences had *Cmm* resistance function reported by Lara-Ávila et al [14].
