# Supplementary material for: In Silico Integrated Analysis of Genomic, Transcriptomic, and Proteomic Data Reveals QTL-Specific Genes for Bacterial Canker Resistance in Tomato (Solanum lycopersicum L.)
Source: Curr Issues Mol Biol. 2023 Feb 6;45(2):1387–95. doi: 10.3390/cimb45020090 (PMC9955012; doi:10.3390/cimb45020090)
Supplement: Supplementary file 1 [file cimb-45-00090-s001.zip › cimb-2162971-supplementary.pdf]

Table S1. Details of studies performed QTL mapping for *Cmm* resistance.

Origin of resistance	Population	Marker system	Bacterial race	QTL method	No. of QTLs	Chromosome	Reference
S. arcanum (LA2157*)*	Three Reciprocal backcross populations	RFLP	*Cmm542*	Kruskal-Wallis rank-sum test	5	T1, T6, T7,T8 and T10	Sandbrink et al. (1995)
S. arcanum LA2157	F2	RFLP-SCAR	*Cmm542*	Kruskal-Wallis rank-sum test	3	T5, T7 and T9	Van Heusden et al. 1999
S. habrochaites LA407	Segregating population derived from IBL	RFLP	*Cmm* A226 and *C290*	Composite interval mapping (CIM)	2	T2 and T5	Coaker and Francis. (2004)
S. pimpinellifolium GI.1554	RIL	SNP	*Cmm*542 unidentified rice from Turkey	Composite interval mapping (CIM)	18	T1, T2, T7, T8 and T12	Sen (2014)

Table S2. Studies identified differentially expressed genes during *Cmm* infection.

Origin of resistance	method	*Cmm* strain	Reference
S. lycopersicum (Rio Grande; Nr mutant and its background lines Pearson and Ailsa Craig)	microarray	*Cmm* 42	Balaji et al. (2008)
S. habrochaites LA2128	cDNA-AFLP	*Cmm* 191	Lara-Ávila, (2012)
S. habrochaites	ESI-MS/MS	C290	Coaker et al (2004)

Table S3. Physical mapping of RFLP probe sequences associated with *Cmm* resistance reported by Sandbrink et al. (1995).

RFLP-probe	Chromosome	% identity	Alignment length	mismatches	gap opens	Probe start	Probe End	Chr. position start	Chr. position end	E-value	Bit score
TG103	T10	99.796	490	1	0	38	527	57572715	57572226	0	900
TG128	T07	100	506	0	0	1	506	62486444	62485939	0	935
TG165	T02	99.376	481	3	0	1	481	35820075	35819595	0	872
TG170	T07	100	522	0	0	1	522	62734671	62735192	0	965
TG174	T07	100	457	0	0	1	457	59483951	59483495	0	845
TG178	T06	97.645	467	4	1	1	467	22112109	22112568	0	795
TG20A	T07	100	502	0	0	1	502	65632413	65632914	0	928
TG210	T07	100	159	0	0	1	159	30282312	30282470	1.96E-78	294
TG223A	T09	100	465	0	0	1	465	2816764	2817228	0	859
TG261	T08	100	491	0	0	1	491	58596385	58596875	0	907
TG307	T08	100	538	0	0	10	547	56177921	56178458	0	994
TG34	T02	100	607	0	0	1	607	49197691	49198297	0	1122
TG35	T09	100	511	0	0	1	511	62387893	62387383	0	944
TG353	T02	99.248	532	4	0	1	532	42997178	42997709	0	961
TG41	T08	99.479	576	0	3	2	574	53832322	53831747	0	1044
TG59	T01	99.07	430	3	1	6	435	77441598	77441170	0	771
TG61	T07	100	412	0	0	2	413	3565619	3566030	0	761
TG9	T09	100	504	0	0	1	504	1942009	1942512	0	931

Table S4. QTLs identified based on physical map of RFLP probes reported by Sandbrink et al. (1995).

QTLs	QTL interval	QTL size (Mb)	Flanking markers
QTL1.1	Single marker	0.00	TG59
QTL2.1	Single marker	0.00	TG165
QTL2.2	42.997178 - 49.197691	6.20	TG353-TG34
QTL7.1	Single marker	0.00	TG61
QTL7.2	Single marker	0.00	TG210
QTL7.3	59.483951-65.632413	6.15	TG174- TG20A
QTL8.1	53.832322 - 58.596385	4.76	TG41-TG261
QTL9.1	1.942009 - 2.816764	0.87	TG9-TG223A
QTL9.2	Single marker	0.00	TG35
QTL10.1	Single marker	0.00	TG103

Table S5. Gene set enrichment analysis of mapped QTLs.

Meta QTLs	chromosome	Resistance origin	Start position (bp)	End position (bp)	Size	No. of genes	No. of genes had known function	% of known function	number of disease resistance genes	Genes had disease resistance function
mQTL2.2	2	*Solanum arcanum*	42997178	49197691	6.2	836	540	64.59	4	(Solyc02g082740.1.1, Solyc02g084610.1.1, Solyc02g084600.4 and Solyc02g084610.1
Rcm2	2	S. habrochaites	49089879	50189289	1.1.	139	124	89.21	0	
Rcm5.1	5	S. habrochaites	59858052	61457155	1.6	140	67	47.86	4	Solyc05g051200.1.1, Solyc05g051310.1.1, Solyc05g050830.3 and Solyc05g050790.3
mQTL7.1	7	*Solanum arcanum*	484118	3565619	3.08	303	213	70.30	11	Solyc07g006700.1.1, Solyc07g007740.1.1, Solyc07g007750.3, Solyc07g007730.4, Solyc07g007735.1, Solyc07g007710.4, Solyc07g007755.1, Solyc07g008373.1, Solyc07g008377.1, Solyc07g008375.1 and Solyc07g006710.2
mQTL7.3	7	*Solanum arcanum*	59483951	65632413	6.15	674	560	83.09	14	Solyc07g056600.1.1, Solyc07g150139.1, Solyc07g053010.3, Solyc07g053020.3, Solyc07g049700.1, Solyc07g052785.1, Solyc07g052800.3, Solyc07g055620.2, Solyc07g055610.3, Solyc07g052770.2, Solyc07g055380.1, Solyc07g052790.3, Solyc07g052780.3, Solyc07g055390.1
mQTL8.1	8	*Solanum arcanum*	53832322	58596385	4.76	435	397	91.26	7	Solyc08g068360.1.1, Solyc08g067380.1, Solyc08g074250.3, Solyc08g075980.2, Solyc08g075630.3, Solyc08g075640.4, Solyc08g076000.4
mQTL9.1	9	*Solanum arcanum*	85353	2816764	2731411	313	282	90.10	8	Solyc09g007010.1.1 and Solyc09g007020.1.1 Solyc09g009240.1.1, Solyc09g005490.1.1, Solyc09g005950.3, Solyc09g006005.1, Solyc09g007010, Solyc09g007020.1

Table S6. Tblastn search of protein sequences had *Cmm* resistance function reported by Lara-Ávila et al (2012).

Gene	chromosome	% identity	alignment length	mismatches	gap opens	Query start	Query end	chr. start	chr. end	e-value	Bit score
ABB90047.1	T00	96.99	133	4	0	1	133	2541421	2541819	7.49E-79	256
BAA10929.1	T00	82.33	266	38	5	1	260	6360040	6359252	1.10E-105	352
CAA63432.1	T01	95.82	383	16	0	1	383	1042254	1041106	0	772
P23322.2	T02	98.82	255	3	0	75	329	34603225	34603989	2.32E-164	511
ABB86276.1	T02	97.87	47	1	0	278	324	48739848	48739988	4.05E-88	95.1
AAD33072.1	T02	94.85	136	7	0	191	326	42723280	42722873	9.66E-135	275
ABY21255.1	T02	83.67	49	7	1	13	61	46721678	46721821	8.57E-78	82
AAA34192.1	T02	79.33	150	2	1	60	180	46730615	46731064	8.61E-84	244
CAK24966.1	T03	97.73	44	1	0	208	251	59914563	59914694	1.31E-135	92
NP_001148439.1	T03	87.64	267	30	1	1	264	547508	548308	1.14E-151	471
BAG80553.1	T03	76.72	451	103	2	5	454	15870044	15871393	0	683
ACH68563.1	T04	90.37	353	4	1	107	429	59981606	59982664	0	610
NP_564149.1	T04	76.19	42	10	0	64	105	2872403	2872528	3.12E-13	71.6
AAO66472.1	T05	100.00	36	0	0	23	58	65017858	65017965	2.99E-15	80.1
ABB72805.1	T06	79.00	100	19	1	245	342	22480227	22480526	1.42E-43	164
AAK52801.1	T07	94.92	59	3	0	365	423	66512336	66512512	5.72E-64	87.4
ACH54085.1	T07	93.75	48	3	0	297	344	24010224	24010367	7.68E-37	95.1
ACG60665.1	T07	89.41	85	9	0	149	233	66168922	66169176	1.06E-25	108
AAB37246.1	T07	85.71	14	2	0	1	14	1671041	1671082	7.72E-137	27.7
BAG16520.1	T08	92.17	115	9	0	963	1077	1439016	1438672	6.47E-87	220
ABC01888.1	T08	85.71	42	6	0	141	182	48263857	48263982	4.21E-32	75.5
EEF34729.1	T08	84.21	95	15	0	606	700	58701556	58701840	8.74E-43	173
Q76CU2.1	T08	62.07	232	57	1	444	644	54718024	54718719	0	305
ACC68681.1	T08	54.67	75	23	3	202	267	52199457	52199239	3.28E-61	75.9
ABK41200.1	T09	97.84	139	3	0	1	139	37242955	37242539	1.38E-71	236
ACC66148.3	T09	94.90	98	5	0	605	702	1549580	1549873	3.62E-50	196
EEF32044.1	T09	94.12	34	2	0	280	313	3438392	3438291	1.44E-26	65.9

Q05538.1	T10	79.54	215	17	1	135	322	56492366	56491722	5.67E-138	348
O49074.1	T10	74.30	393	23	5	118	432	64515144	64513966	1.44E-178	558
P32980.1	T12	89.20	250	25	1	1	248	62527570	62528319	6.66E-122	385
